# Association between pubic hair grooming and prevalent sexually transmitted infection among female university students

**DOI:** 10.1371/journal.pone.0221303

**Published:** 2019-09-04

**Authors:** Jamie Luster, Abigail Norris Turner, John P. Henry, Maria F. Gallo

**Affiliations:** 1 Division of Epidemiology, College of Public Health, Ohio State University, Columbus, Ohio, United States of America; 2 Division of Infectious Diseases, College of Medicine, Columbus, Ohio, United States of America; 3 Clinical Health Division, Columbus Public Health, Columbus, Ohio, United States of America; University of the Pacific, UNITED STATES

## Abstract

Recent findings have suggested an association between pubic hair grooming and self-reported history of sexually transmitted infection (STI), specifically gonococcal infection (GC), chlamydial infection (CT), or human immunodeficiency virus (HIV). We evaluated the association between self-reported extreme grooming and laboratory-confirmed prevalence of GC/CT. Between April 2017 and April 2018, we enrolled English-speaking, adult, female students at a large, Midwestern university who presented on-campus for STI testing. Participants completed a questionnaire on demographics and sexual and grooming behaviors, which was linked to their GC/CT test results based on nucleic acid amplification testing. We defined extreme grooming as removal of all pubic hair either at least weekly in the past 12 months or ≥6 times in the past 30 days. We used two separate logistic regression models to determine whether odds of GC/CT varied by extreme groomer status for either time interval. In the study sample of 214 women, prevalence of GC/CT was 9.8%. Nearly all participants (98.1%) reported ever grooming; 53.6% were extreme groomers in the past year and 18% in the past month. Extreme grooming was not associated with prevalent GC/CT in the past year (odds ratio [OR] = 0.8; 95% confidence interval [CI]: 0.3–1.9; adjusted OR = 0.7; 95% CI: 0.3–2.0) or in the past month (OR = 0.5; 95% CI: 0.1–2.0; aOR = 0.4; 95% CI: 0.1–1.9). Pubic hair grooming was common among female university students attending for STI testing. Findings do not support pubic hair grooming as an STI risk factor in this population.

## Introduction

Sexually transmitted infections (STIs) are prevalent in the U.S. and can have lifelong consequences if untreated [1]. Gonococcal (GC) and chlamydial infection (CT) are the most common STIs in the U.S., and their rates have been increasing since 2013. Young women 15–24 years of age have the highest incidence of GC and CT compared to other sex and age groups in the U.S. [1]. In 2017, the rate of GC infection was 557.4 per 100,000 women 15–19 years of age and 684.8 per 100,000 women 20–24 years of age. The corresponding CT rates were 3,265.7 and 3,985.8 per 100,000 women 15–19 years and 20–24 years of age, respectively [2]. Risk factors for STI among young adults include high number of sexual partners or high sexual frequency, identifying as a racial, ethnic, or sexual minority, experiencing forced or transactional intercourse, or being incarcerated, or of low socioeconomic status [1,3,4].

A recent analysis suggested a possible association between pubic hair grooming and self-reported history of STI, specifically a combined outcome of GC, CT, or human immunodeficiency virus (HIV) [5–7]. Pubic hair grooming, defined as trimming or removal of some or all hair in the genital region, is common in both women and men in the U.S. [8,9]. Grooming is most prevalent among young, white, adult women with high levels of income and education [8]. The potential mechanism of exposure between pubic hair grooming and GC, CT, and HIV is unknown. Pubic hair grooming could lead to increased STI risk via the sharing of grooming tools with an infected individual; however, fomite transmission of GC or CT has never been documented [7]. Grooming–especially frequent grooming and removal of all pubic hair–can cause injury [10]. The process of stripping away pubic hair could cause microtrauma in the skin’s mucocutaneous barrier, thereby facilitating pathogen entry and transmission [11]. Alternatively, the observed relationship between grooming and STI could be due to confounding. For example, individuals who groom more frequently might have more sex, thereby resulting in greater exposure to STI. Furthermore, young age is associated with risk of STI as well as with pubic hair grooming [1,4,7].

## Materials and methods

Our study objective was to evaluate the association between self-reported extreme grooming and laboratory-confirmed prevalence of GC/CT among female university students presenting at one of two walk-in STI testing sites on the campus of a large, Midwestern university from April 2017 to April 2018. Eligible study participants were female, at least 18 years of age, spoke and read English, and presented for GC/CT testing. Using an electronic tablet with REDCap [12], participants completed a questionnaire on demographics, sexual behavior, STI risk factors, and pubic hair grooming. The questions were adapted from previous research to maximize comparability of results [6]. Only women who provided written consent were enrolled into the study, and The Ohio State University Biomedical Institutional Review Board approved the research.

### Independent variable

Our main exposure was a binary yes/no variable for *extreme grooming within the past 12 months*. Extreme grooming was defined as responding either “Weekly” or “Daily” to the question, “In the past 12 months, which best describes how often you have removed all of your pubic hair?” Non-extreme groomers were defined as participants who responded “Monthly,” “Less than 12 times,” or “Never.” As a sensitivity analysis, we used an alternative dichotomous variable for *extreme grooming in the past 30 days* based on responding either “11+ times” or “6–10 times” to “In the past 30 days, how often have you removed all of your pubic hair?” Non-extreme groomers for this measure were those who responded "2–5 times,” “1 time,” or “Never.”

### Dependent variable

Urine samples for GC/CT testing were collected at study sites by trained test counselors affiliated with the university and were sent to a nearby health department for processing and testing using the Aptima Combo 2® assay (Hologic, Marlborough, MA). We linked STI test results with questionnaire records using unique participant study identification numbers. We created a binary variable to describe whether a participant tested positive for GC/CT (either or both infections vs. neither).

### Confounders

**Fig 1:**
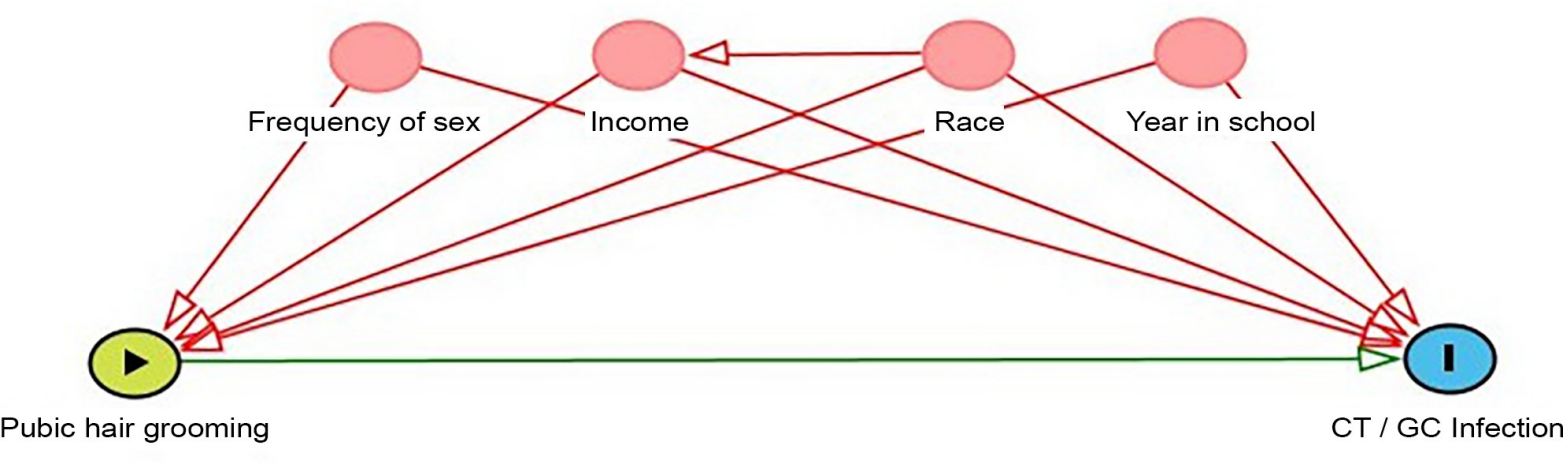
Directed acyclic graph. The minimally sufficient adjustment set includes the variables frequency of sex, income, race, and year in school.

Possible correlates of both pubic hair grooming and STI were identified based on existing literature [3,4,7,8,13] and through the creation of a directed acyclic graph (DAG) (Fig 1). The DAG illustrates correlates that are potential confounders of the relationship between grooming and STI, that is, the variables that were associated with both the exposure and the outcome but were not on the hypothesized causal pathway. Potential confounders were sexual frequency, parent/guardian income, current year in university, and race.

### Analysis

We calculated descriptive statistics for all demographic variables (i.e., race, parental/guardian income, year in school, relationship status, and sexual orientation and identity), sexual activity and grooming. We used separate logistic regression models to determine whether GC/CT status varied by extreme groomer status 1) within the past 12 months or 2) within the past 30 days. We used manual backward selection by adding all variables identified as potential confounders from the DAG (Fig 1) (i.e., sexual frequency, parental/guardian income, grade level, and race) to the regression model, removing them one at a time, and then examining whether their removal changed the magnitude of the resulting odds ratio for the association between grooming and STI. If a variable changed the odds ratio 10% or more, it was retained in the model as a confounder. We also fit a full model that adjusted for all confounders identified in directed acyclic graph. Sexual frequency was divided into three categories: weekly or more often, less often than weekly but more often than monthly, and monthly or less often. Annual parental/guardian income was categorized as less than $30,000, $30,000 to less than $60,000, $60,000 to less than $100,000, or $100,000 or greater. For year in school, respondents were categorized by year (1^st^ through 5^th^ or greater). We created three categories for race: white, Black, and other, including Asian, American Indian, Native Hawaiian, more than one race, and those who selected “other” race. The sample size was insufficient to stratify race into more distinct categories. We used SAS (Statistical Analysis System; version 9.4; SAS Institute; Cary, NC) to conduct all analyses.

## Results and discussion

### Participants

Overall, 249 female students enrolled in the study. We excluded participants who completed the questionnaire but failed to complete STI testing (n = 10) and those for whom we were unable to link questionnaire and STI test results (n = 18). The inability to link data could have resulted from participants providing different names to study staff and STI testing staff or from participants leaving the site before giving a specimen for STI testing. Finally, we excluded participants with testing encounter dates that differed from their questionnaire date (n = 4) and those who failed to complete the questionnaire (n = 3). The final analysis population consisted of the remaining 214 participants.

In a sensitivity analysis, we used exact chi-squared tests for categorical variables and Wilcoxon rank-sum tests for continuous variables to compare demographic characteristics and sexual behaviors of participants with STI data versus those who were excluded from the analysis for missing STI data (S1 Table). Only one factor statistically significantly differed between the two groups: participants included in the analysis had a mean of 4.3 partners (SD = 4.0) while excluded participants had a mean of 3.4 partners (SD = 3.6; p = 0.03).

Most women in the analysis population were white (75.2%), single (72.0%), and reported a parental or guardian income of $60,000 or greater (81.4%; Table 1). The mean age of participants was 20.7 years (standard deviation = 1.7). Nearly all respondents had ever had vaginal, oral, or anal sex (99.5%). Prevalence of GC/CT infection was 9.8% in the study population. Two participants tested positive for GC, 19 tested positive for CT, and none tested positive for both infections. Nearly all participants reported ever grooming their pubic hair in their lifetime (n = 209, 98.1%).

**Table 1:** Demographic characteristics and sexual behaviors among female university students attending for sexually transmitted infection testing.

	All women (n = 214)		Extreme groomer in past month (n = 38)		Extreme groomer in past year (n = 112)	
	No.	(%)	No.	(%)	No.	(%)
Year in college						
1^st^ year	41	(19.3)	7	(18.4)	20	(17.9)
2^nd^ year	58	(27.2)	12	(31.6)	36	(32.1)
3^rd^ year	45	(21.1)	11	(29.0)	25	(22.3)
4^th^ year	39	(18.3)	8	(21.1)	21	(18.8)
5^th^ year or higher	30	(14.1)	0		10	(8.9)
Missing	1		0		0	
Annual parental/guardian income						
<$30,000	10	(5.2)	1	(3.0)	2	(1.9)
$30,000 to <$60,000	26	(13.4)	3	(9.1)	10	(9.6)
$60,000 to <$100,000	61	(31.4)	13	(39.4)	32	(30.8)
≥$100,000	97	(50.0)	16	(48.5)	60	(57.7)
Missing	20		5		8	
Race						
White	161	(75.2)	31	(81.6)	89	(79.5)
Black	27	(12.6)	3	(7.9)	8	(7.1)
Other	26	(12.2)	4	(10.5)	15	(13.4)
Relationship status						
Single	154	(72.0)	28	(73.7)	88	(78.6)
Dating	52	(24.3)	9	(23.7)	20	(17.9)
Engaged	2	(0.9)	0	(0.0)	2	(1.8)
Other	6	(2.8)	1	(2.6)	2	(1.8)
No. sexual partners in past 12 mos.^a^						
Mean, standard deviation	4.3	4.0	4.0	2.3	5.0	4.8
Missing	7		1		2	
Ever had anal, vaginal, or oral sex						
Yes	210	(99.5)	38	(100.0)	111	(100.0)
No	1	(0.5)	0	(0.0)	0	(0.0)
Decline or missing	3		0		1	
Sex of past partner(s)						
Men	181	(86.6)	37	(97.4)	101	(91.8)
Women	5	(2.4)	0		2	(1.8)
Both	23	(11.0)	1	(2.6)	7	(6.4)
Decline or missing	5		0		2	
Sexual frequency^a^						
Daily to weekly	84	(40.4)	14	(36.8)	48	(43.2)
<Weekly and >monthly	64	(30.8)	15	(39.5)	37	(33.3)
Monthly or less often	60	(28.9)	9	(23.7)	26	(23.4)
Missing	6		0		1	
Age of most recent sex partner^a^						
Mean, standard deviation	21.7	2.8	21.4	2.7	21.4	2.2
Missing	6		0		1	
Sex while drunk or high in past year^a^						
Yes	165	(79.0)	31	(81.6)	94	(84.7)
No	44	(21.0)	7	(18.4)	17	(15.3)
Decline or missing	5		0		1	
Condom use during vaginal sex in past month						
Always	36	(16.8)	6	(15.8)	16	(14.3)
Never	46	(21.5)	9	(23.7)	26	(23.2)
Inconsistent	104	(48.6)	20	(52.6)	59	(52.7)
No vaginal sex in past month	28	(13.1)	3	(7.9)	11	(9.8)
Gonococcal infection						
Yes	2	(0.9)	0		0	
No	212	(99.1)	38	(100.0)	112	(100.0)
Chlamydial infection						
Yes	19	(8.9)	2	(5.3)	10	(8.9)
No	195	(91.1)	36	(94.7)	102	(91.1)
Gonococcal or chlamydial infection						
Yes	21	(9.8)	2	(5.3)	10	(8.9)
No	193	(90.2)	36	(94.7)	102	(91.1)

^a^ Among those reporting ever vaginal, oral or anal sex.

Among those who reported ever grooming, 112 (53.6%) were extreme groomers within the past year, defined as removal of all pubic hair weekly or daily within the past 12 months. Thirty-eight (18.0%) participants were extreme groomers within the past month, defined as removal of all pubic hair 6 or more times within the past 30 days. Most respondents reporting using a non-electric blade razor to groom most often (82.9%), either with soap (41.7%) or with shaving cream (41.2%). Nearly two-thirds (63.3%) had ever had a grooming injury, and the mean number of lifetime grooming injuries was 4.9 (SD = 3.8).

In both unadjusted and adjusted models, we found no evidence of difference in the odds of GC/CT between women who were extreme groomers within the past year compared to those who were not extreme groomers (OR = 0.8, 95% CI = 0.3–1.9; adjusted OR (aOR) = 0.6, 95% CI = 0.3–2.0, respectively; Table 2). Unadjusted and adjusted associations between extreme grooming within the past month and prevalent GC/CT were somewhat stronger but remained non-significant (unadjusted OR = 0.5, 95% CI = 0.1–2.0; aOR = 0.4, 95% CI = 0.1–1.9; Table 2).

**Table 2:** Association between extreme grooming and gonorrhea or chlamydial infection among female university students attending for sexually transmitted infection testing (n = 214).

	Unadjusted		Reduced model^c^		Full model^d^	
Pubic grooming	OR	(95% CI)	aOR	(95% CI)	aOR	(95% CI)
Extreme, past 12 months^a^						
No	1.0		1.0		1.0	
Yes	0.8	(0.3–1.9)	0.7	(0.3–2.0)	0.7	(0.2–1.9)
Extreme, past 30 days^b^						
No	1.0		1.0		1.0	
Yes	0.5	(0.1–2.0)	0.4	(0.1–1.9)	0.4	(0.1–2.0)

aOR = adjusted odds ratio; CI = confidence interval; OR = odds ratio

^a^ Defined as removal of all pubic hair weekly or daily in past 12 months.

^b^ Defined as removal of all pubic hair 6 or more times in past 30 days.

^c^ OR for past 12 months was adjusted for sexual frequency and parental/guardian income. OR for past 30 days was adjusted for race.

^d^ Adjusted for all confounders identified in directed acyclic graph: annual parental/guardian income, year in school, race, and sexual frequency.

We found no evidence of an association between extreme pubic hair grooming (in the past 12 months or the past 30 days) and prevalent GC or CT infection among a convenience sample of female, mostly white, single, university students presenting for STI testing at a large Midwestern university. In contrast, three previous studies have suggested a link between these practices and prevalent STI. First, a case series of 30 individuals presenting with new cases of sexually transmitted molluscum contagiousum virus at a dermatology office in France showed that 93% of infected individuals practiced pubic hair grooming (70% shaving, 13% clipping, 10% waxing) [5]. However, this analysis had a small sample size, did not test for more prevalent STIs including chlamydia and gonorrhea, and had no comparison group. Second, a cross-sectional survey of 4,062 men found an increased prevalence of self-reported genital infections and abscesses with higher frequency of grooming among those who reported having sex with men compared to men who have sex with women or compared to non-groomers. The analysis did not adjust for potential confounders and temporality could not be established due to the study design [6].

Most recently, a large web survey of men and women 18–65 years of age found that 74% of study participants reported ever having groomed their pubic hair [7]. Among those who had groomed, 17% were extreme groomers (defined as removing all pubic hair more than 11 times within the past year) and 22% were “high-frequency” groomers (defined as engaging in daily or weekly pubic hair trimming). Overall, 7% of respondents reported a history of secretory STI (defined as CT, GC, or HIV). After adjustment for age and lifetime number of sexual partners, ever groomers had nearly two times higher odds of self-reported history of secretory STI when compared to never groomers; similar effects were observed comparing extreme and high-frequency groomers to non-groomers. These findings were limited by potential for introduction of bias from using self-reported STI as the outcome. Additionally, the use of lifetime STI as the outcome does not allow for temporal approximation of risk due to grooming. Finally, the analysis only controlled for age and lifetime number of sexual partners, and the observed association could be the result of residual confounding [7].

Our analysis is an important contribution to the scant literature about this topic for several reasons. First, we used laboratory-confirmed GC/CT to measure STI status rather than rely on respondent reporting. Self-reported history of CT infection has been shown to be have only moderate agreement with serologically-confirmed infection [14]. Our analysis also collected detailed information on potential confounders, which allowed us to compute adjusted estimates using multivariable regression models. Study weaknesses included the small sample size, which led to the imprecision evident in the wide confidence intervals for measures of association. Also, we enrolled a convenience sample of women at a single university who were attending for STI testing; the findings may not be applicable to a more general population.

## Conclusions

In summary, ever pubic hair grooming was almost universal (98.1%) in our sample, which is consistent with previous estimates of grooming prevalence among college-aged individuals [9]. However, in contrast to earlier studies, we found no association between frequent grooming and STI prevalence. Our findings do not support for the need for public health or clinical interventions to address pubic hair grooming as a risk factor for prevalent GC or CT. Future studies on this topic could use a larger, more representative sample to allow for more precise estimates and wider generalizability. Future research also could be strengthened with mixed methods forms of data collection to permit a thorough understanding of participant behaviors related to grooming practices.

## Supporting information

S1 Table Demographic characteristics and sexual behaviors among female university students attending for sexually transmitted infection (STI) testing for those with and without STI data. (DOCX)
