# Supplementary material for: Association between pubic hair grooming and prevalent sexually transmitted infection among female university students
Source: PLoS One. 2019 Sep 4;14(9):e0221303. doi: 10.1371/journal.pone.0221303 (PMC6726136; doi:10.1371/journal.pone.0221303)
Supplement: S1 Table — (DOCX) [file pone.0221303.s001.docx]

| **S1 Table. Demographic characteristics and sexual behaviors among female university students attending for sexually transmitted infection (STI) testing for those with and without STI data.** | | | | | |  |
| --- | --- | --- | --- | --- | --- | --- |
|  | Participants with STI data  (n=214) | | Participants missing STI data  (n=32) | | p-value^b^ |  |
|  | No. | (%) | No. | (%) |  |  |
| Year in college |  |  |  |  |  |  |
| 1^st^ year | 41 | (19.3) | 4 | (12.5) | 0.51 |  |
| 2^nd^ year | 58 | (27.2) | 9 | (28.1) |  |  |
| 3^rd^ year | 45 | (21.1) | 9 | (28.1) |  |  |
| 4^th^ year | 39 | (18.3) | 8 | (25.0) |  |  |
| 5^th^ year or higher | 30 | (14.1) | 2 | (6.3) |  |  |
| Annual parental/guardian income |  |  |  |  |  |  |
| <$30,000 | 10 | (5.2) | 1 | (3.4) | 0.76 |  |
| $30,000 to <$60,000 | 26 | (13.4) | 5 | (17.2) |  |  |
| $60,000 to <$100,000 | 61 | (31.4) | 6 | (20.7) |  |  |
| ≥$100,000 | 97 | (50.0) | 17 | (58.6) |  |  |
| Race |  |  |  |  |  |  |
| White | 161 | (75.2) | 23 | (74.2) | 0.95 |  |
| Black | 27 | (12.6) | 5 | (16.1) |  |  |
| Other | 26 | (12.2) | 3 | (9.7) |  |  |
| Relationship status |  |  |  |  |  |  |
| Single | 154 | (72.0) | 19 | (61.3) | 0.47 |  |
| Dating | 52 | (24.3) | 11 | (35.5) |  |  |
| Engaged | 2 | (0.9) | 0 | (0.0) |  |  |
| Other | 6 | (2.8) | 1 | (3.2) |  |  |
| No. sexual partners in past 12 mos.^a^ |  |  |  |  |  |  |
| Mean, standard deviation | 4.3 | 4.0 | 3.4 | 3.6 | 0.03 |  |
| Ever had anal, vaginal, or oral sex |  |  |  |  |  |  |
| Yes | 210 | (99.5) | 29 | (96.7) | 0.23 |  |
| No | 1 | (0.5) | 1 | (3.3) |  |  |
| Sex of past partner(s) |  |  |  |  |  |  |
| Men | 181 | (86.6) | 24 | (82.8) | 0.66 |  |
| Women | 5 | (2.4) | 0 | (0.0) |  |  |
| Both | 23 | (11.0) | 5 | (17.2) |  |  |
| Sexual frequency^a^ |  |  |  |  |  | |
| Daily to weekly | 84 | (40.4) | 9 | (31.0) | 0.47 | |
| <Weekly and >monthly | 64 | (30.8) | 12 | (41.4) |  | |
| Monthly or less often | 60 | (28.9) | 8 | (27.6) |  | |
| Age of most recent sex partner ^a^ |  |  |  |  |  | |
| Mean, standard deviation | 21.7 | 2.8 | 21.5 | 2.0 | 0.95 | |
| Sex while drunk or high in past year ^a^ |  |  |  |  |  | |
| Yes | 165 | (79.0) | 21 | (72.4) | 0.64 | |
| No | 44 | (21.0) | 8 | (27.6) |  | |
| Condom use during vaginal sex in past month |  |  |  |  |  | |
| Always | 36 | (16.8) | 7 | (24.1) | 0.56 | |
| Never | 46 | (21.5) | 9 | (31.0) |  | |
| Inconsistent | 104 | (48.6) | 12 | (41.4) |  | |
| No vaginal sex in past month | 28 | (13.1) | 1 | (3.4) |  | |
| ^a^Among those reporting ever vaginal, oral or anal sex.  ^b^ From exact chi-squared tests for categorical variables and Wilcoxon rank-sum tests for continuous variables. | | | | | | |
